# Raw eye tracking data of healthy adults reading aloud words, pseudowords and numerals

**DOI:** 10.1016/j.dib.2023.109360

**Published:** 2023-07-02

**Authors:** Marco Pedrotti, Anne-Françoise de Chambrier, Paolo Ruggeri, Jasinta Dewi, Myrto Atzemian, Catherine Thevenot, Catherine Martinet, Philippe Terrier

**Affiliations:** a Haute Ecole Arc Santé, HES-SO University of Applied Sciences and Arts Western Switzerland, Espace de l'Europe 11, Neuchâtel 2000, Switzerland; b Haute Ecole Pédagogique Vaud, Lausanne, Switzerland; c Brain Electrophysiology Attention Movement Laboratory, Institute of Psychology, University of Lausanne, Switzerland; d Laboratory of Brain and Cognitive Development, Institute of Psychology, University of Lausanne, Switzerland; e Department of Thoracic and Endocrine Surgery, University Hospitals of Geneva, Switzerland

**Keywords:** Reading, Numbers, Numerals, Words, Pseudowords, Eye movements, Eye tracking, EyeLink 1000

## Abstract

This paper describes data from de Chambrier et al. (2023). The dataset [2] contains raw eye tracking data of 36 healthy adults, collected using an EyeLink 1000 (SR Research Ltd., ON, Canada) during an on-screen reading task. Participants read 96 items including words, pseudowords and numerals. Each item was presented at the center of the screen until the participant produced an oral response and pressed the keyboard's space bar.

Part of the data were analyzed to extract key metrics such as fixation number, fixation duration, saccade number, and saccade amplitude identified by the EyeLink 1000 [1]. Reuse potential includes (but is not limited to) pupil diameter data analysis, identification of fixations and saccades using custom algorithms, and secondary analyses using participant demographics (age, gender) as independent variables.

**Specifications Table**

**Table:** 

Subject	Experimental and Cognitive Psychology
Specific subject area	Reading
Type of data	Eye tracking raw data sampled at 1000 Hz using an EyeLink 1000 (SR Research Ltd., ON, Canada)
How the data were acquired	The items were presented at the center of a 24-inch LCD monitor (visible screen width and height: 520 × 325 mm) with a resolution of 1’920 × 1’200 (refresh rate: 60 Hz). The dominant eye-gaze position was recorded at a sampling frequency of 1000 Hz with a desktop-mounted EyeLink 1000 eye tracker, placed at 530 mm distance in front of the participant.
Data format	Raw
Description of data collection	Each trial included the sequential presentation of a target item and a blank screen. The target item was presented on the screen until the participant produced an oral response and pressed the space bar. Immediately following the response, a blank screen was presented for 100 ms, followed by the drift check indicating the beginning of the next trial.
Data source location	• Institution: University of Lausanne, Géopolis • City/Town/Region: Lausanne • Country: Switzerland • Latitude and longitude (and GPS coordinates, if possible) for collected samples/data: Latitude: 46.526556; Longitude: 6.579237
Data accessibility	Repository name: Zenodo Data identification number: 7962917 Direct URL to data: https://doi.org/10.5281/zenodo.7962917[2] Instructions for accessing these data: Just click on the doi link above
Related research article	de Chambrier, A. F., Pedrotti, M., Ruggeri, P., Dewi, J., Atzemian, M., Thevenot, C., Martinet, C., & Terrier, P. (2023). Reading numbers is harder than reading words: An eye-tracking study. Acta Psychologica, 237, 103942. https://doi.org/10.1016/j.actpsy.2023.103942[1]

## Value of the Data

• Reproducibility: Other researchers could reproduce the results and verify our scientific claims.
• These data can be useful for researchers interested in reading (e.g., developmental psychologists, neuropsychologists), as well as for people interested in event (fixations, saccades, microsaccades, blinks, and so forth) detection from raw eye tracking samples (e.g., computer scientists, neuroscientists, and so forth).
• Possible data use/reuse cases include (but are not limited to) pupil diameter data analysis, identification of fixations and saccades using custom algorithms, as well as secondary analyses (e.g., using participant demographics as independent variables). Pseudowords were precisely matched to words, and eye movements that occurred on these two categories of items could also be compared.

## 1 Objective

Reading numbers is a skill required in daily life and is fundamentally different compared to reading words. Word reading follows a phonographic system, in which graphic units (e.g. letters, syllables) do not have a meaning on their own but by being assembled with other symbols to form words. Number reading follows a logographic (or ideographic) system, in which each symbol (e.g. 1, 5, 0) has a meaning in itself but can be further combined to express quantities larger than 9. During the last four decades, eye movements have allowed to generate extensive knowledge on the cognitive processes occurring while reading text, however we could not find research that targeted possible eye movement differences when reading words compared to numbers. We generated this dataset to spot such differences.

This data article adds to the published article (a) a detailed description of the raw data publicly available in a data repository and (b) some suggestions on how these data could be used/reused.

## 2 Data Description

Table 1 contains the names of variables in the dataset. For further details, see http://sr-research.jp/support/files/dvmanual.pdf.

**Table 1:** Names of variables in the dataset.

TRIAL_INDEX	Unique trial identifier (1 to 96)
LEFT_GAZE_X	Left eye gaze coordinate along the x axis (pixels)
LEFT_GAZE_Y	Left eye gaze coordinate along the y axis (pixels)
LEFT_PUPIL_SIZE	Left eye pupil size (in arbitrary units) of the current sample
RIGHT_GAZE_X	Right eye gaze coordinate along the x axis (pixels)
RIGHT_GAZE_Y	Right eye gaze coordinate along the y axis (pixels)
RIGHT_PUPIL_SIZE	Right eye pupil size (in arbitrary units) of the current sample
TIMESTAMP	The time stamp of the sample (in milliseconds since EyeLink tracker was activated
TrialTextShown	The stimulus (word, pseudoword or numeral) currently shown on screen

*Note*: LEFT_GAZE_X, LEFT_GAZE_Y, LEFT_PUPIL_SIZE are empty if the recorded eye is the right one. RIGHT_GAZE_X, RIGHT_GAZE_Y, RIGHT_PUPIL_SIZE are empty if the recorded eye is the left one.

## 3 Experimental Design, Materials and Methods

### 3.1 Participants

Participants were 36 students from the University of Lausanne psychology course (27 women and 9 men; mean age = 21.3 years old; SD = 4.15). All had normal or lenses-corrected vision, no history of learning disorders, and were native French speakers. Table 2 contains age and gender of each participant.

**Table 2:** Participants demographics.

Participant number	Age (years)	Gender
1	18	female
2	unknown	male
3	22	female
4	21	female
5	23	female
6	19	female
7	22	female
8	21	female
9	23	male
10	21	female
11	21	female
12	20	female
13	19	male
14	43	male
15	19	female
16	23	female
17	21	female
18	19	male
19	23	female
20	21	female
21	22	female
22	22	female
23	18	male
24	18	female
25	19	female
26	25	female
27	21	male
28	22	female
29	19	female
30	22	female
31	19	female
32	20	female
33	22	male
34	19	male
35	19	female
36	20	female

### 3.2 Procedure

Participants read 96 items in total. They were individually placed in a quiet, low-lit room, seated 930 mm from the PC screen. We presented items at the center of a 24-inch LCD monitor with visible screen width and height of 520 × 325 mm, resolution of 1′920 × 1′200 px and refresh rate of 60 Hz. Table 3 contains all the stimuli displayed. A chin and forehead rest ensured a correct head position. We determined eye dominance using the hole-in-card test with participants' hands and centered gaze. Gaze position of the dominant eye was measured at a sampling rate of 1000 Hz with a table-mounted EyeLink 1000 positioned 530 mm in front of the participant.

Each trial was preceded by a drift check, during which the experimenter ensured that the participant centered his or her gaze within a black circle 0.48° in diameter and then validated the start of the trial. Then began the sequential presentation of the target item displayed on a gray background, until the participant generated a spoken response and pressed the space bar on the keyboard. Immediately after the response, a blank screen was displayed for 100 ms, followed by the drift check that signaled the start of the next trial. We presented target items in two blocks of trials: One block involved the presentation of 48 randomly displayed numeral trials, and the other block involved the presentation of 24 randomly displayed word trials followed by 24 randomly displayed pseudoword trials. We counterbalanced the order of the two blocks across participants. Before each category of items, we asked participants to read the items out loud as accurate and quickly as possible. For the pseudoword category, we indicated that the items had no meaningfulness.

**Table 3:** Stimuli.

	Words	Pseudowords	Numerals with separators	Numerals without separators
**Short**	amer	érul	1′549	1293
	aula	fabu	2′391	1362
	brut	inor	2′497	2638
	déjà	iqué	3′165	3127
	écho	isan	3′618	3748
	être	muar	4′976	4579
	fuir	oufé	5′482	5862
	iris	spac	6′578	6124
	saga	stau	6′814	6925
	très	udre	7′849	7524
	unir	ujar	8′743	8597
	user	zago	9′736	9351

**Long**	astucieux	birtajicer	19′582′743	24739165
	baptiser	carriloge	2′137′954′867	57468391
	boulangerie	daurinfarue	23′914′856′297	76951238
	caoutchouc	frinchar	327′569′184	175928346
	carrelage	lomanube	39′681′742′538	624731958
	dorénavant	ostéciant	43′829′516	938426157
	franchir	outigieux	536′287′419	3158963472
	fréquemment	pannesquier	6′178′249′358	4863167295
	impatient	pondaser	7′849′125′463	8627514359
	limonade	pouitcheau	82′745′961′354	16259874317
	participer	quimmévrant	857′491′326	37862951462
	questionner	tulanévont	97′483′261	97654328164

## Ethics Statements

This study was approved by the ethical committee of the University of Lausanne (reference of decision: C_SSP_022021_00006). The research was carried out in accordance with the Declaration of Helsinki. All participants signed an informed consent. They received 2 points to validate a methodology course as well as a 15 CHF voucher for a bookshop.

## CRediT authorship contribution statement

**Marco Pedrotti:** Conceptualization, Methodology, Validation, Data curation, Writing – original draft, Visualization, Supervision. **Anne-Françoise de Chambrier:** Conceptualization, Methodology, Investigation, Writing – original draft, Supervision. **Paolo Ruggeri:** Software, Validation, Investigation, Data curation, Writing – original draft. **Jasinta Dewi:** Investigation, Writing – review & editing. **Myrto Atzemian:** Investigation, Writing – review & editing. **Catherine Thevenot:** Resources, Supervision, Writing – review & editing. **Catherine Martinet:** Conceptualization, Supervision, Writing – review & editing. **Philippe Terrier:** Data curation, Methodology, Validation, Formal analysis, Writing – original draft, Visualization.

## Declaration of Competing Interest

The authors declare that they have no known competing financial interests or personal relationships that could have appeared to influence the work reported in this paper.

## Data Availability

EyeLink 1000 raw eye tracking data - Reading numbers is harder than reading words: An eye-tracking study (Original data) (Zenodo).
